# Strengthening global commitment to eliminating cervical cancer: What lessons from the past can we apply to the future?

**DOI:** 10.7189/jogh.10.020385

**Published:** 2020-12

**Authors:** Katayoun Taghavi, Misinzo Moono, Serra Asangbeh, Grant Gillett, Margaret Pascoe, Albert Manasyan

**Affiliations:** 1 Institute of Social and Preventive Medicine, University of Bern, Switzerland; 2 The Graduate School for Cellular and Biomedical Sciences of the University of Bern, Bern Switzerland; 3 Centre for Infectious Disease Research in Zambia, Lusaka, Zambia; 4 Department of Bioethics, University of Otago, Dunedin, New Zealand; 5 Auckland University of Technology, University of Auckland, New Zealand; 6 Newlands Clinic, Harare, Zimbabwe; 7 University of Alabama at Birmingham, Alabama, USA

Cervical cancer (CC) is largely preventable, however, while some high-income countries are nearing elimination status, it remains the leading cause of cancer-related death among women in many low- and middle-income countries (LMIC) [1]. Simms et al showed that with widespread coverage of both HPV vaccination and effective cervical screening by 2020, between 12.5 to 13.4 million cases of CC could be averted by 2070 [2]. This would, in turn, reduce CC incidence to less than four cases per 100 000, and effectively “eliminate” this disease by the end of the century, irrespective of development indices. Today, 85% of women diagnosed with cervical cancer live in LMIC [3] and the striking differences in access to vaccines, screening tests, diagnostic equipment and treatment between high- and low-income countries cannot be ignored. Though not the sole requirement for effective implementation of cancer prevention programs, without these, urgent scale-up required to achieve elimination in some countries will not be feasible.

## LEARNING FROM PAST HEALTHCARE REFORM

We believe that the success of past coordinated efforts to improve access to antiretroviral therapy (ART) provides a model we can draw upon to address the present inequities in diagnosing and treating CC. In the early 2000s, 95% of people with HIV/AIDS lived in developing countries [4]. An estimated 6 million people were in immediate need of ART yet only around 300 000 were receiving it [4,5]. At this time, a strong climate of global public health reform arose in response to the inequities in access to health care between low- and high-income countries. At the 4th Ministerial Conference of the World Trade Organization (WTO) in Doha in November 2001, 142 countries adopted a historic condition on the TRIPS Agreement and Public Health. The “Doha declaration” recognized that while innovation and development is critical for medical advancement and requires protection, access of medicines among vulnerable populations should be paramount. The Doha declaration allowed the production of generic versions of patented antiretroviral (ARV) medicines increasing affordability in low-resource settings. It is considered a landmark in global health care policy that placed public health needs above commercial interests in international trade negotiations. The increase in access to ART that followed enabled the transition of HIV/AIDS in sub-Saharan Africa from a terminal illness to a chronic one. While the Doha declaration has not been a panacea for the inequities in health care access between low- and high-income countries, its impact on the HIV/AIDS epidemic provides a compelling case study for today’s global health issues and the discrepancies that exist. Similar efforts are now required to curb the unnecessary mortality arising from CC.

**Figure:**
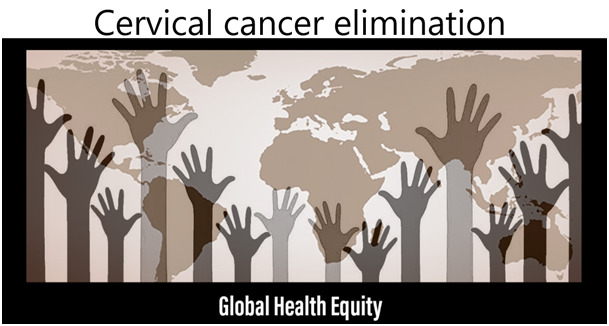
Photo: Internationally, progress towards cervical cancer elimination is unequal. This image was developed by the authors for this manuscript.

## GLOBAL INTERVENTIONS AS THE FOUNDATION FOR REFORM

The WHO initiative to eliminate cervical cancer is an excellent starting point demonstrating consensus at a global level to address this public health problem [6]. So far, three interim targets are identified as necessary for simultaneous implementation: 90% of girls should be fully vaccinated with the HPV vaccine by the age of 15; 70% of women should be screened using a high-performance test by 35 and 45 years of age; and 90% of women identified with neoplastic disease should be treated. Countries that have implemented HPV vaccination programmes alongside high quality screening and diagnostics for CC have seen a dramatic decline in CC incidence [7]. In contrast, similar testing and treatment is rarely emulated in LMIC. Economic constraints, including the cost of vaccines and diagnostic equipment in laboratories and clinics, resulting in reduced access to treatment are barriers to effective cervical screening for vulnerable women in these settings. Often cervical cancer screening strategies must be negotiated in a delicate health care infrastructure, with a scarcity of specialized health care workers, and competing health priorities, and these issues also contribute to this continuing inequality [8,9]. Unless disparities in access to vaccines, diagnostics and treatments are addressed, the necessary scale-up required in some countries may be impracticable ^,^[10]. This is recognized in the draft global strategy which specifies the requirement for equitable access in the context of universal health care and effective approaches towards resource mobilization.

To reduce the global burden of CC, access to vaccines, diagnostics and treatment modalities must be improved. These measures, would in turn, assist in strengthening the shrinking health care force in LMICs which is, and will be under considerable strain in the pandemic era. First, the supply of HPV vaccines must be accelerated, and they must be affordable in non-GAVI countries. The current shortage in supply means that many countries have to postpone implementation programs [11]. Furthermore, achieving a price of less than $5 (not including operational costs) per fully vaccinated girl to ensure affordability in LMIC, requires a multi-sectoral approach [12]. Second, efforts to replace visual inspection with acetic acid (VIA) with more accurate screening methods are urgently required and the affordability of HPV point-of-care testing warrants further evaluation. Third, acceptable treatment must be linked with care. Though treatment for pre-cancerous lesions are largely available, African countries remain well below the WHO’s recommendations for radiotherapy services [13] and the availability of analgesic medicines are also limited, with only 11 out of 47 African countries using morphine for pain relief [14]. This presents a significant problem as pain is the most common presenting symptom of women presenting with cervical neoplasia [15].

One mechanism to reduce prices for essential health products is through licensing agreements between patent holders and local generic producers. Compulsory licensing is a mechanism under the TRIPS agreement that allows a government body to grant a license to an entity other than a patent holder, allowing them to produce the patented product in exchange for adequate remuneration [16]. We encourage civil society, private entities, and LMIC governments to form a strong coalition to push for the use of compulsory licensing to improve the availability of low-cost generic HPV vaccines, diagnostics, and treatments without risk of trade retaliation [11].

At the global level, there is evidence that the inequities in access to health care in the non-communicable diseases (NCDs) era, and specifically relating to CC, are being acknowledged [17]. The WHO has initiated a campaign to increase awareness and initiate action from member states to prioritize vaccination and screening programs. Interim targets for synchronized primary and secondary prevention strategies have been established. The relationship between these objectives and trade agreements, TRIPS Agreement (including the Doha amendment), has been acknowledged in Article 36 of the recent Political Declaration for the high-level meeting on NCDs adopted in October 2018 [18]. Notably, Article 38 explicitly states that access to affordable diagnostic, screening, treatment and care, as well as vaccines are integral to a comprehensive approach to prevention and control of all NCDs [18]. Operationalizing these sentiments requires international co-operation, transparency and accountability. We call for countries to address extending the Doha declaration to cervical cancer screening at the TRIPs council within the World Trade Organization. At a national level, we urge countries to strengthen their national legal framework to facilitate vaccination, screening and treatment and engage with the private sector to safeguard compliance with article 36. Cooperation between Member states and the private sector is essential to promote the recent UNGA Political declaration on the prevention and control of NCDs adopted in October 2018. Furthermore, to ensure equitable access to health care services for all, LMIC governments need to develop national health care financing policies that include adequate legislative, technical and regulatory frameworks. We recommend that financing of cancer control be adequately allocated from national health care budgets supplemented by innovative financing mechanisms, particularly in countries where rising incomes are enabling expansion of public financing for health.

## CHALLENGES AND OPPORTUNITIES BROUGHT BY THE COVID-19 PANDEMIC

In the aftermath of the COVID-19 pandemic, there will be challenges and opportunities for eliminating CC. A potentially large proportion of health resources and funding in LMIC will again be siphoned away from women’s health and cervical screening, yet CC will remain a pressing health concern. Other challenges include: (i) unwillingness to access the health facilities for vaccination or routine CC screening due to fear of COVID-19 exposure; (ii) inability to access health facilities due to transportation disruptions or overall restriction of movement due to lockdown; (iii) disruption to CC screening campaigns in communities due to risk of exposure during group meetings; (iv) disruption of school-based vaccinations due to their closure; (v) health care providers may treat patients differently if they feel unsafe due to lack of appropriate protective equipment, perpetuating further unwillingness of patients to seek medical care; (vi) inability to access the screening facility due to facility restrictions and a focus on essential health care provision only. However, on the positive side is an increasing capacity for and prioritization of treatments for patients diagnosed with precancerous and cancerous lesions. Increasing access to protective gear, vaccines, diagnostics and treatments would assist in strengthening the shrinking health care force in LMICs under considerable strain during the COVID-19 pandemic.

## CONCLUSION

A multisectoral approach is required in order to address the global challenges of CC prevention, especially in the aftermath of the Covid-19 pandemic where health resources will be strained further. The WHO initiative to eliminate cervical cancer is an excellent starting point to achieve these goals at the global level. Economic incentives are also critical to back up these good intentions, and there are lessons we can learn from the reform of ART access in the HIV/AIDS context to be applied in an era of non-communicable disease. The health care sector must work with policy makers and industry to promote equitable access to resources including life-saving vaccines, diagnostic equipment, and treatments and protective equipment. Global health priorities need to address the increasing burden of cervical cancer, which disproportionately affect LMIC, and issues of financing and access must be addressed.
